# Implementing a disease management standard operating procedure in a contemporary private pediatric dental practice

**DOI:** 10.3389/fdmed.2026.1781479

**Published:** 2026-04-30

**Authors:** Katelyn Whitlatch, Carson Richardson, Samuel Cocke, Jonny Norris, Beau Meyer

**Affiliations:** 1 Division of Pedaitric Dentistry, The Ohio State University College of Dentistry, Columbus, OH, United States; 2 The Ohio State University Center for Biostatistics, Columbus, OH, United States; 3 Montshire Pediatric Dentistry, Keene, NH, United States

**Keywords:** dental caries classification, minimally invasive dentistry, pediatric dentistry, sealants, silver diamine fluoride, stainless steel crowns

## Abstract

**Introduction:**

Dental caries is a prevalent childhood condition with significant health and economic consequences. Disease management techniques, such as silver diamine fluoride (SDF) and sealants, have emerged as alternatives to traditional restorative care. This study describes the implementation of a disease management protocol in a large, multi-site private pediatric dental practice.

**Methods:**

A retrospective descriptive cohort analysis was conducted using de-identified electronic health record data from Montshire Pediatric Dentistry at one of its nine locations between August 2018 and June 2025. Children with at least two visits were included. Visits and procedures were categorized by caries subtype and treatment type. Escalation was defined as progression from disease management to higher-intensity interventions including restoration or other significant treatment. Descriptive statistics formed the basis of analysis.

**Results:**

Among 8,857 children, 41.9% were caries-free. Disease management procedures dominated treatment types, with SDF and sealants most common. Among children with caries, 46% had treatment or disease management initiated at their initial visit, and 35% experienced a higher-intensity treatment escalation over the duration of care, primarily in severe subtypes (Grade III and V). Median time to higher-intensity treatment escalation was approximately 547 days, and median duration of care from initial to final treatment was 637 days.

**Conclusion:**

Disease management procedures can be successfully implemented in private practice. Many children treated at this practice had treatment initiated at their first practice visit, and higher-intensity treatments occurred nearly a year and a half after initial treatment.

## Introduction

Dental caries is a common childhood condition, and its consequences if left untreated can range from pain, infection, difficulties eating, and disrupted sleep, to time away from school, hospitalization, and even death in extreme cases (1–6). The odds of having dental caries and untreated decay is significantly higher for children from low-income households, rural areas, or those with Medicaid insurance (7, 8). When Medicaid enrollees get dental treatment, the disease is often in advanced stages, requiring more expensive dental treatments (9, 10).

In recent years, disease management treatments for dental caries have increased (11), shifting the treatment paradigm towards arresting disease and preserving dentition. Some evidence suggests that this approach can delay the need for advanced and expensive procedures such as sedation or general anesthesia (12–14). In 2018, the American Dental Association (ADA) published clinical practice guidelines for nonrestorative treatment of caries lesions supporting disease management procedures including silver diamine fluoride (SDF) application, sealants, and resin infiltration (15). In 2023, the ADA published clinical practice guidelines for restorative treatment of caries lesions, again prioritizing minimally invasive techniques following selective caries removal (16). Neither guideline explicitly states when nonrestorative vs. restorative approaches should be used. Ultimately, practices and patients make these decisions.

A large, multi-site, private pediatric dental practice in the northeast United States has implemented disease management procedures as its standard operating procedure. The primary purpose of this study was to characterize the implementation of disease management in pediatric dentistry at one of their clinical sites. Specifically, this analysis described different types of disease management procedures provided to different subtypes of dental caries, including how often higher-intensity treatment escalated beyond the initial treatment.

## Materials and methods

This retrospective descriptive cohort study was reviewed by the Ohio State University Institutional Review Board (STUDY 2025E0150) and determined to be exempt under exemption category #4b. A data use agreement was executed with the private practice to access de-identified data.

### Study context

Montshire Pediatric Dentistry is a private practice operating in 9 locations across 3 states in the northeast United States. Each site has a different payor mix ranging from 46% to 90% Medicaid, but each site follows the same standard operating procedure for treatment planning and clinical decision making. The standard operation procedure is prevention-driven and includes separate decision algorithms for primary and permanent teeth. The providers in this study followed the same decision tree algorithm when providing treatment (See Supplementary File). Data from this study were obtained from the original, single practice location, but the operating procedures were applied at all locations.

### Implementation process

The practice used in this study had 3 dentists. Two of these dentists authored the standard operating procedures (one graduated dental school in 2010 and the other in 2015), and the third provider joined in 2021 (graduated dental school in 2017). The new provider trained during onboarding to follow standard treatment planning protocols and algorithms. The new provider calibrated on the algorithms via case discussions with the owner partners during the first 90 days of hire and continued mentorship throughout employment.

Non-cavitated lesions were treated with a combination of SDF, dental sealant, glass ionomer cement, or fluoride varnish. Cavitated lesions were treated by selective caries removal followed by SDF plus glass ionomer cement [defined as silver-modified atraumatic restorative technique (SMART) for this study], SDF plus stainless steel crown (SSC) using Hall technique, or only SSC using Hall technique. Infected or necrotic teeth were planned for extraction. Restoration type was predicated on the severity or size of the presenting lesion. However, parents were given autonomy to select a treatment option for their child. After a discussion of the risks, benefits, and prognosis, some parents elected a less invasive treatment despite more invasive treatment recommended by a provider. In these instances, the less invasive option was provided with an abbreviated follow-up period (e.g. 4–12 weeks) to monitor or retreat as needed

### Data collection

Visit-level data from August 16, 2018, through June 30, 2025 was collected from Montshire Pediatric Dentistry's electronic health record (EHR), Open Dental Software (Salem, OR). The patient variables of interest included a unique patient identifier, sex, age in years and months at the first completed visit within the study window, number of days between each visit, tooth-level treatments for each permanent and primary tooth, any supernumerary tooth treatments, any diagnostic and preventative Current Dental Terminology (CDT) codes used, use of nitrous oxide, use of general anesthesia, and total dental charges (i.e., billed amounts) for treatment completed on a given date. All patient-level identifiers were anonymized by removing direct identifiers prior to transferring data to the study team, and each patient record was assigned a randomized, unique ID to ensure the protection of patient confidentiality. Children with fewer than 2 clinic visits were excluded from analysis since they could not be evaluated on the study outcomes.

Custom structured query language queries were written to extract these variables. The queries were developed and refined in coordination with Open Dental's technical support team to ensure alignment with the intended definitions of the variables and to follow the best practices for accessing data directly from the system. To ensure accuracy of data collection, data extraction underwent several validation steps including query testing and refinement, random audits, and internal reviews. The queries were iteratively tested and improved based on sample outputs and feedback from the support team. Subsets of collected data were randomly selected and compared against the patient charts within the EHR to confirm that the extracted information matched the source records. Where discrepancies were identified, adjustments were made to the queries to improve the accuracy before the final data sets were delivered. No exclusion criteria were applied to the data queries.

### Variable definitions

First, each clinic visit was categorized into one of three visit types: diagnostic, preventive, or treatment. Visits were categorized using a hierarchical classification approach, which included diagnostic visits as procedure codes D0000 to D0999; preventive visits as D0000 to D1999 (excluding D1354); and treatment visits as D1354, D2000 to D3999 and D7140.

Second, subjects were categorized based on their disease status and subtype to provide important context about disease management procedures. Previous work using large clinical databases identified six distinct subtypes of early childhood caries to predict caries in the permanent dentition (17). In the current study, the subtypes defined by Gormley et al. (17) were applied by using a combination of visit types and CDT codes. The subtypes were assigned individually per child irrespective of time. The subtypes included:

- Grade 0 or caries free: only having diagnostic (D0000 to D0999) or preventive visits (D0000 to D1999, excluding D1354)
  1. No history of treatment visits; assumed that children who received only dental sealants (D1351) received them as primary prevention rather than as treatment for dental caries
- Grade I (pit and fissures of molars): having a treatment visit (D1354, D2000 to D3999, or D7140)
  1. Any of the treatment codes on any teeth #A,J,K,L,S,T
- Grade II (smooth surface of anterior teeth): having a treatment visit (D1354, D2000 to D3999, or D7140)
  1. Any of the treatment codes on any teeth #D,E,F,G
- Grade III (posterior teeth plus central incisors): having a treatment visit (D1354, D2000 to D3999, or D7140)
  1. Any of the treatment codes on any teeth #A,B,E,F,I,J,K,L,S,T
- Grade IV (posterior teeth plus all maxillary incisors): having a treatment visit (D1354, D2000 to D3999, or D7140)
  1. Any of the treatment codes on any teeth #A,B,D,E,F,G,I,J,K,L,S,T
- Grade V (lesions involving the canines): having a treatment visit (D1354, D2000 to D3999, or D7140)
  1. Any of the treatment codes on any teeth #A,B,C,D,E,F,G,H,I,J,K,L,M,R,S,T

While the current study was not designed to predict caries lesions in the permanent dentition as in the cited study (17), the subtype classification scheme helped organize this descriptive analysis.

Third, to provide more detail about the types of treatment provided to each child, treatment visits were further classified as disease management, restoration, or significant treatment visits. Disease management was restricted to procedure codes D1351, D1354, and D2940; restoration was D2331 to D2335, D2390 to D2394, and D2930 to D2931; and significant treatment as D3110, D3120, D3220, D7140, and D7210. These definitions were introduced to identify when disease management strategies were escalated to higher-intensity treatments beyond the initial treatment. It is possible that children in the caries free group received dental sealants as part of disease management rather than purely prevention; however, this scenario is unlikely based on the decision tree in the standard operating procedure.

Last, each treatment visit was defined as a higher-intensity escalation if the treatment rendered on a particular tooth increased from disease management to restoration, restoration to significant treatment, or disease management to significant treatment. The intent was to capture when a tooth transitioned from a less severe disease state to a more severe state across clinic visits. Escalation was recorded as yes vs. no and as the number of days from the initial treatment to a higher-intensity treatment. To reiterate, escalation was tracked through tooth-level procedure sequencing, while caries subtypes were assigned using visit types and procedure codes rather than formal diagnostic calibration. Time from initial exam to first treatment and final treatment was calculated as the number of days from a patient's first diagnostic or preventive visit to their first treatment visit and final treatment visit. If the initial exam and treatment occurred on the same day, the time was recorded as zero.

### Statistical analysis

Summary statistics for continuous variables were described as means and standard deviations (SD) or medians and interquartile range (IQR), and categorical variables as frequencies (*n*) and percentages (%). All analyses were performed using R statistical software version 4.5.0 (R Core Team, 2025; Vienna, Austria).

## Results

Out of 10,910 children that were assessed in the initial query, 8,857 had at least 2 clinic visits in the study period and were included in analysis. Of these children, 3,709 (41.9%) were caries free (Table 1). Among the caries subtypes, the most severe subtypes were among the most common (Grade III: 28.2%, Grade V: 11.9%). The mean age at first visit was youngest for Grade II (mean = 2.5y, SD = 1.9y) and oldest for Grade I (mean = 6.5y, SD = 3.7y).

**Table 1:** Descriptive summary of treatment rendered under a disease management protocol in a private pediatric dentistry practice treating 8,857 children with at least 2 visits between August 16, 2018, through June 30, 2025. Results are stratified by early childhood caries subtypes defined by Gormley et al. (17).

Characteristic	Caries Free *N* = 3,709	Grade I *N* = 1,220	Grade II *N* = 118	Grade III *N* = 2,495	Grade IV *N* = 260	Grade V *N* = 1,055	Overall *N* = 8,857
Age at first visit (years), mean (SD)	6.9 (5.7)	6.5 (3.7)	2.5 (1.9)	5.4 (2.5)	3.2 (2.2)	6.1 (3.1)	6.1 (4.4)
Use of nitrous oxide (n, % yes)	243 (7%)	214 (18%)	10 (8%)	525 (21%)	44 (17%)	362 (34%)	1,398 (16%)
Time from initial exam to first treatment (days)	-	113 (0, 604)	332 (98, 630)	29 (0, 469)	167 (0, 442)	30 (0, 299)	42 (0, 469)
Time from initial exam to final treatment (days)	-	434 (13, 1,013)	383 (98, 741)	690 (188, 1,264)	952 (494, 1,446)	706 (210, 1,295)	637 (128, 1,218)
Treatment completed at initial visit (n, % yes)	-	484 (40%)	18 (15%)	1,258 (50%)	86 (33%)	507 (48%)	2,353^a^ (46%)
Escalation to higher-intensity Intervention Required (n, % yes)	-	125 (10%)	2 (1.7%)	1,045 (42%)	119 (46%)	530 (50%)	1,821^a^ (35%)
Time to higher-intensity intervention (days)	-	552 (201, 1,008)	294 (141, 446)	534 (217, 1,008)	686 (286, 1,066)	547 (202, 1,002)	547 (217, 1,008)
Total dental charges from initial exam to final treatment (USD)	-	1,131 (693, 1,800)	578 (432, 888)	1,694 (1,009, 2,625)	1,886 (1,201, 2,680)	1,912 (1,039, 3,016)	1,546 (893, 2,505)
Disease Management Procedures (median (IQR))	-	4 (1, 9)	4 (2, 7)	13 (7, 19)	17 (9, 25)	13 (4, 23)	4 (0, 12)
Restorative Procedures (median (IQR))	-	0 (0, 0)	0 (0, 0)	1 (0, 2)	0 (0, 3)	1 (0, 3)	0 (0, 1)
Significant Treatment Procedures (median (IQR))	-	0 (0, 0)	0 (0, 1)	0 (0, 0)	0 (0, 2)	0 (0, 2)	0 (0, 0)

SD, standard deviation; USD, United States dollar; IQR, Interquartile range.

^a^ Only among those with caries treatment procedures.

Proportionally, nitrous oxide use was highest for children with Grade V subtypes (*n* = 362, 34%). General anesthesia was used for 31 children, 15 with Grade V subtypes (1.4%) and 13 from Grade III subtypes (0.5%). Disease management procedures dominated the treatment procedure definitions. The median number of disease management procedures ranged from 4 (IQR: 1, 9) for Grade I subtypes to 17 (IQR: 9, 25) for Grade IV subtypes; whereas the median number of restorative procedures ranged from 0 for Grade I subtypes to 1 (IQR: 1, 3) for Grade V subtypes (Table 2). Less severe subtypes were predominantly treated with sealants and SDF, whereas more severe subtypes had treatment including sealants, SDF, SMART technique, SSC, and extractions. Forty-six percent of children with caries were able to receive treatment at their initial visit.

**Table 2:** Procedure counts among 8,857 children treated in a private pediatric dental practice utilizing a disease management protocol.

Dental treatment type	Summary statistic	Caries free (*N* = 3,709)	Grade I(*N* = 1,220)	Grade II(*N* = 118)	Grade III(*N* = 2,495)	Grade IV (*N* = 260)	Grade V(*N* = 1,055)	Overall (*N* = 8,857)
Dental sealant	Mean (SD)	1.1 (2.5)	2.2 (2.9)	1.7 (3.0)	2.5 (3.0)	2.7 (2.9)	1.7 (2.6)	1.8 (2.8)
	Median (IQR)	0.0 (0.0)	0.0 (5.0)	0.0 (0.0)	0.0 (6.0)	2.0 (5.0)	0.0 (3.0)	0.0 (4.0)
	Range	0.0–14.0	0.0–10.0	0.0–7.0	0.0–14.0	0.0–14.0	0.0–9.0	0.0–14.0
SDF	Mean (SD)	0.0 (0.0)	2.8 (2.7)	2.3 (2.4)	9.9 (7.2)	11.6 (7.0)	11.4 (10.0)	4.9 (7.3)
	Median (IQR)	0.0 (0.0)	2.0 (3.0)	2.0 (4.0)	9.0 (10.0)	12.0 (10.2)	9.0 (14.5)	1.0 (8.0)
	Range	0.0–0.0	0.0–19.0	0.0–8.0	0.0–50.0	0.0–36.0	0.0–55.0	0.0–55.0
SMART	Mean (SD)	0.0 (0.0)	0.7 (1.5)	0.1 (0.5)	1.3 (2.4)	3.1 (3.8)	1.8 (3.1)	0.8 (2.0)
	Median (IQR)	0.0 (0.0)	0.0 (1.0)	0.0 (0.0)	0.0 (2.0)	2.0 (5.0)	0.0 (2.0)	0.0 (0.0)
	Range	0.0–0.0	0.0–10.0	0.0–4.0	0.0–21.0	0.0–17.0	0.0–26.0	0.0–26.0
Crown	Mean (SD)	0.0 (0.0)	0.2 (0.6)	0.0 (0.0)	1.2 (1.6)	1.3 (1.8)	1.7 (2.0)	0.6 (1.4)
	Median (IQR)	0.0 (0.0)	0.0 (0.0)	0.0 (0.0)	1.0 (2.0)	0.0 (2.0)	1.0 (3.0)	0.0 (0.0)
	Range	0.0–0.0	0.0–6.0	0.0–0.0	0.0–8.0	0.0–7.0	0.0–8.0	0.0–8.0
Extraction	Mean (SD)	0.0 (0.0)	0.2 (0.5)	0.4 (0.7)	0.3 (0.7)	1.1 (1.7)	1.1 (1.6)	0.3 (0.9)
	Median (IQR)	0.0 (0.0)	0.0 (0.0)	0.0 (1.0)	0.0 (0.0)	0.0 (2.0)	0.0 (2.0)	0.0 (0.0)
	Range	0.0–0.0	0.0–3.0	0.0–4.0	0.0–6.0	0.0–8.0	0.0–9.0	0.0–9.0

Results are stratified by early childhood caries subtypes defined by Gormley et al. (17).

Treatment escalated to higher-intensity interventions for 20.6% of the total sample, and 35% of the children with dental caries. The greatest proportion of these were among the most severe subtypes, (Grade III: 42%, Grade V: 50%) and procedurally, treatment escalation was lowest for crowns (92 crowns escalated to extractions, 1.7% of all crowns placed). For these children, the median time to higher-intensity intervention was nearly 550 days after initial treatment (Grade III: 534 days, IQR: 217, 1,008; Grade V: 547 days, IQR: 202, 1,002).

Similarly, mean dental charges were greatest for the most severe subtypes (Grade V mean = $2,177, SD = $1,424). The median number of days from initial exam to first treatment was lowest for Grade III (median = 29, IQR: 0, 469) and Grade V (median = 30, IQR: 0, 299), meaning that for severe subtypes, treatment was initiated very soon after initial presentation. The median number of days from initial exam to final treatment was more variable, but averaged 637 days overall (IQR: 128, 1,218).

## Discussion

This study showed how disease management techniques were implemented as a standard operating procedure in a private pediatric dental practice setting. Disease management procedures, such as SDF and SMART technique restorations, were the overwhelming treatments delivered to children with dental caries. One in three children experienced a higher-intensity intervention following initial disease management interventions; however, this escalation to a higher-intensity intervention occurred approximately 550 days after the initial treatment. More simply, when disease management procedures required subsequent higher-intensity intervention, they did so nearly a year and a half after initial treatment. The median duration from initial to final treatment was 637 days, likely reflecting reapplications of SDF. This extended period may also represent a meaningful window of child development. This duration could allow a child to transition from negative to positive behavior, thereby delaying more invasive restorative treatment to a later stage when the child is better able to accept it, and the treated teeth are still functional at that age. The median charges over this nearly 2-year period were $1546. These findings, though entirely descriptive, provide an example for how disease management techniques, as a standard operating procedure, can be successfully implemented and scaled within a private pediatric dental practice.

### Comparison to existing literature

SDF is a highly effective treatment for caries arrest in the primary dentition, with caries arrest estimates ranging from 54% to 88% (15, 18, 19). Within a community-based practice, SDF-treated teeth had one-year survival rates around 75% (20). In a large, private insurance dental claims study, 40% of SDF-treated primary teeth received additional treatment, such as restoration or extraction, over a 2-year period (21). Teeth that received additional treatment were more likely posterior teeth and teeth expected to be in the mouth more than 2 additional years (21). In one clinical trial comparing SDF to restorations, SDF-treated teeth had significantly more minor failures than restored teeth at 1-year, emphasizing the need for careful monitoring, follow-up, and reapplication with SDF treatment (22). The current study aligns with existing literature in that, through careful monitoring and follow-up over a long period, 35% of children received higher-intensity treatments following initial SDF application. Children with more severe dental caries subtypes (Grade III, IV, and V) had a higher frequency of treatment escalation, but the escalation occurred more than a year and half after initial treatment. Extrapolating the finding, these subtypes are ones where disease management techniques may be less successful at the technical level but can be important adjuncts to the overall treatment plan when considering the child's family and social situation as well as systemic access to care barriers (i.e., long waitlists for surgical rehabilitation).

In this study, less than 2% of the SSC, primarily placed via Hall technique, required treatment escalation to extraction. As a proxy for survival rates, this escalation to higher-intensity intervention percentage compares favorably to previously reported success of Hall technique (92% to 96% 2-year survival rates across 2 studies) and traditional SSCs (91% to 93% 2-year survival rates across 2 studies) (23). Placing SSCs via Hall technique offers significant advantages over traditional techniques by requiring shorter chair times, not requiring local anesthesia, and being more comfortable; all features that align well with a disease management protocol.

### Strengths and limitations

The main strength of this descriptive study was the addition of a caries subtype classification to contextualize future treatment within a disease management framework. Even though our application of the subtyping was treatment code driven as ever receiving treatment during the study window, its use provided important operational details on procedures and treatment sequencing. The use of pragmatic, private practice data provided more real-world data than if this study were conducted at an academic medical center. However, this type of data made it difficult to control diagnosis calibration, case selection, and additional demographic descriptions to complement the analysis. The practice's use of a standard operating procedure reduces some, but not all, of these concerns.

A significant limitation was reproducibility at practices or locations outside of the Montshire Pediatric Dentistry network and the generalizability of the findings. While additive to the overall reporting, study variables and outcome definitions posed a limiting factor on reproducibility. Escalation to higher-intensity intervention was defined by sequences of tooth-level procedure codes, whereas caries subtypes were assigned retrospectively using visit types and procedure codes rather than assigned at the initial visit based on formal diagnostic calibration. Another limitation was that the decision to escalate to a higher-intensity treatment may not have been directly related to disease progression or treatment failures. Treatment escalation to higher-intensity interventions may have resulted from planned staging of care, the patient's behavioral readiness, payer factors, or the clinician's decision making. Additionally, data collection relied on the accuracy of the custom structured query language queries. Although validation methods ensured that data collection was accurate, the data were collected from an EHR, not directly from the subjects.

## Conclusion

Disease management as a standard operating procedure can be successfully implemented in a pediatric private practice setting. For severe dental caries subtypes, these techniques serve as important interim treatments until teeth can be restored to form, function, and esthetics. This model was applied irrespective of the insurance payers, though some policies may limit the full-scale implementation of procedures such as SDF (24). Based on the patient volumes in this study, minimally invasive disease management techniques are generally an acceptable treatment philosophy for treating dental caries in a private practice setting. Further research is needed to develop an in-depth understanding of the parent or guardian's value perception of this method of care as well as to develop an understanding of the difference between the direct costs of a disease management model compared to traditional models of care.

## Data Availability

The datasets presented in this article are not readily available because the data was only made available to the research team through a data use agreement. Requests to access the datasets should be directed to Beau Meyer, meyer.781@osu.edu.
