# Supplementary material for: Implementing a disease management standard operating procedure in a contemporary private pediatric dental practice
Source: Front Dent Med. 2026 Apr 30;7:1781479. doi: 10.3389/fdmed.2026.1781479 (PMC13171848; doi:10.3389/fdmed.2026.1781479)
Supplement: Supplementary file 1 [file Datasheet1.pdf]

Montshire Dental Policy and Procedure

Policy Name: Treatment Planning

PURPOSE:

To establish a standard of care and provide a framework to educate team members regarding Montshire Dental clinical treatment planning philosophies.

POLICY/PROCEDURE:

Montshire Dental aims to provide the best care in the world utilizing the latest scientific literature and techniques to craft a treatment plan that is individualized to each child's specific needs.
We want to deliver on the following goals:

1.) Help children have a positive view towards dentistry.
2.) Prevent and avoid dental pain and loss of teeth.
3.) Create a healthy oral environment for the adult dentition to prevent and treat dental disease/ disease progression.
4.) Accomplish #1-#3 in as minimally invasive way as possible; exhaust the least invasive methods first.

In addition to the above, a successful outcome is informed by reducing the emergency dental visits and reducing the need for sedation dentistry in the communities we serve.

Montshire Dental utilizes both medical and surgical means to accomplish the purpose listed above.
Education is emphasized in every patient encounter to encourage individuals to take good care of their families and live healthier lives.
Parents/patients are to be given all treatment options including no treatment relevant to their situation – however – some treatment options do not fit in with our philosophy (i.e. zirconia crowns).
Treatment planning recommendations are given in the context of thorough explanation and rationale.

Prevention

-Caries etiology is to be explained to all patients in a manner imitating the diagram below:

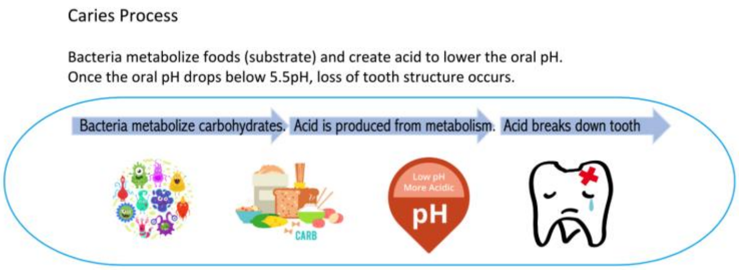

-Over the counter fluoride toothpaste is recommended for children: grain of rice size amount until age 3, green pea size amount age 3+ years.
-Prescription toothpaste is recommended for high-risk children who are able to spit out toothpaste reliably.
-Home use of povidone iodine is recommended to prevent and treat dental caries/ gingivitis. Directions for use are placed in every operatory; patients/parents are recommended to take a photo for future implementation.
-Glass ionomer sealants are recommended for primary and permanent teeth due to their antimicrobial and remineralization properties.
- All recommendations are based on scientific evidence/ literature.

Recommendations for posterior baby teeth: see the diagram below.
The boxes indicate the relevant treatment options in line with Montshire Dental philosophy.
Note: Class II RMGI restorations are typically not recommended due to limited success rate.

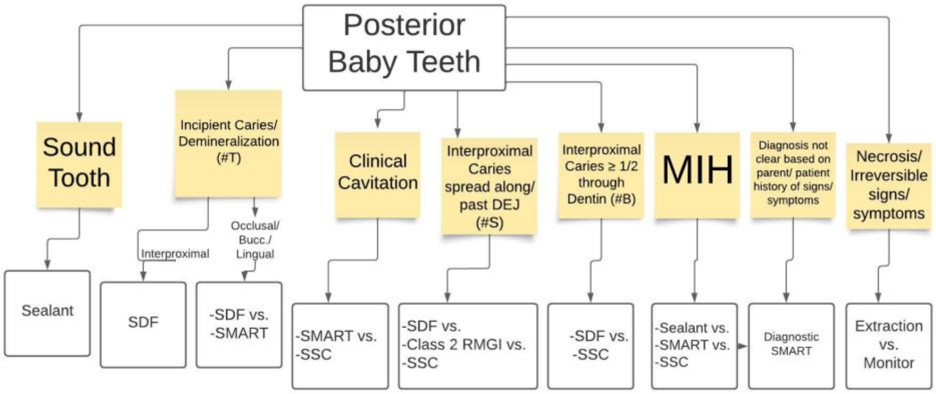

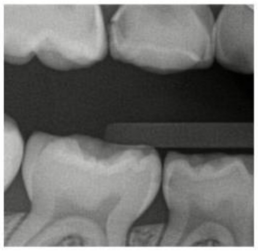

Recommendations for anterior baby teeth:

- For caries, we typically recommend Povidone Iodine – to avoid staining in the esthetic region of the mouth.
- Good OH and adequate use of fluoride is recommended to prevent progression & necrosis.
- Recommendations regarding necrotic teeth: when there is pain/swelling – typically recommend extraction. Draining teeth with no pain can be monitored or extracted depending on parent/patient preference. By not treating an abscess, damage may occur to the permanent dentition.
- As always, parents need to be educated regarding all tx options including: restorations, crowns, etc. – and can be referred if desired.

Recommendations for posterior adult teeth: see the diagram below.

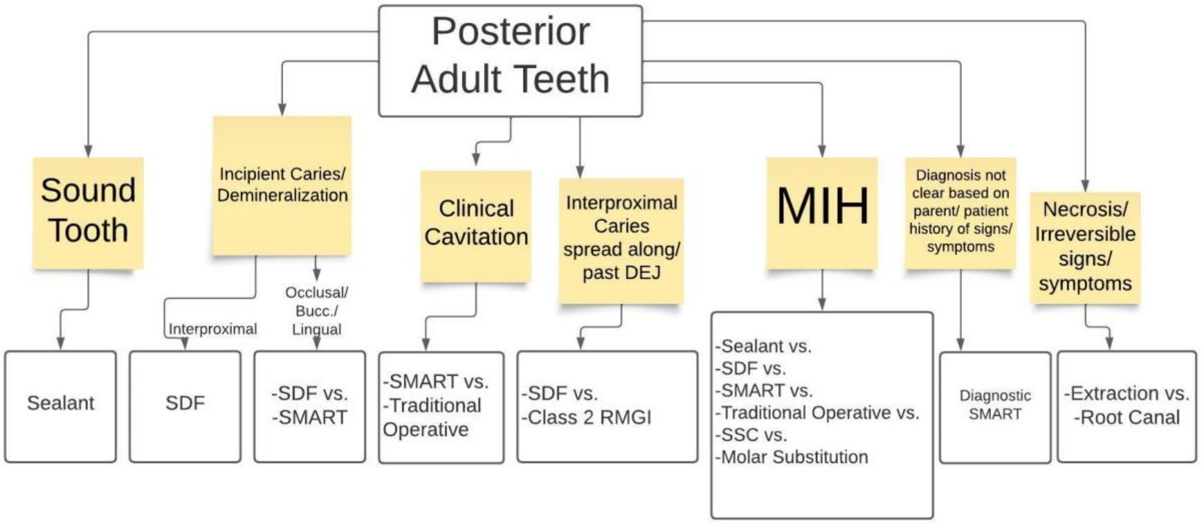

American Dental Association Caries Classification System (Young D, Nový B, Zeller G et al. The American Dental Association Caries Classification System for Clinical Practice. *J Am Dent Assoc*. 146, 79-86.)

- A helpful tool for education, discussion, and calibration.

	Sound	Initial	Moderate	Advanced
**Clinical Presentation**	No clinically detectable lesion. Dental hard tissue appears normal in color, translucency, and gloss.	Earliest clinically detectable lesion compatible with mild demineralization. Lesion limited to enamel or to shallow demineralization of cementum/dentin. Mildest forms are detectable only after drying. When established and active, lesions may be white or brown and enamel has lost its normal gloss.	Visible signs of enamel breakdown or signs the dentin is moderately demineralized.	Enamel is fully cavitated and dentin is exposed. Dentin lesion is deeply/ severely demineralized.
**Other Labels**	No surface change or adequately restored	Visually noncavitated	Established, early cavitated, shallow cavitation, microcavitation	Spread/disseminated, late cavitated, deep cavitation
**Infected Dentin**	None	Unlikely	Possible	Present
**Appearance of Occlusal Surfaces (Pit and Fissure)***	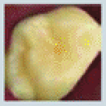	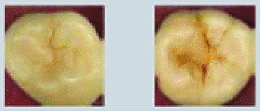	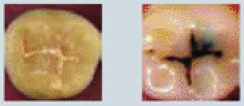	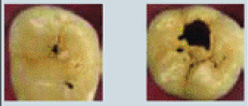
**Accessible Smooth Surfaces, Including Cervical and Root†**	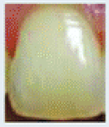	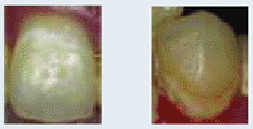	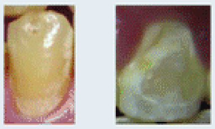	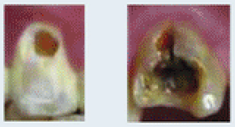
**Radiographic Presentation of the Approximal Surface‡**	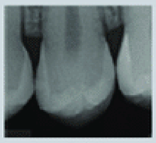 E0§ No radiolucency	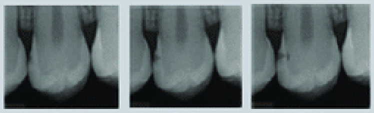 E1§ E2§ D1§ Radiolucency may extend to the dentinoenamel junction or outer one-third of the dentin. Note: radiographs are not reliable for mild occlusal lesions.	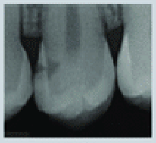 D2§ Radiolucency extends into the middle one-third of the dentin	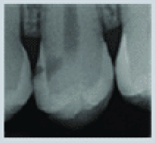 D3§ Radiolucency extends into the inner one-third of the dentin

* Photographs of extracted teeth illustrate examples of pit-and-fissure caries.
† "Cervical and root" includes any smooth surface lesion above or below the anatomical crown that is accessible through direct visual/tactile examination.
‡ Simulated radiographic images.
§ E0-E2, D1-D3 notation system.[33]

E1/E2/D1 = typically recommend SDF
D2/D2 = SDF vs. Traditional Restoration

- Variables include: caries risk, patient preference, access to care, cooperation, etc.
- Consider O-ring placement to open contact and observe clinically in 1 week & SDF application if non-cavitated.

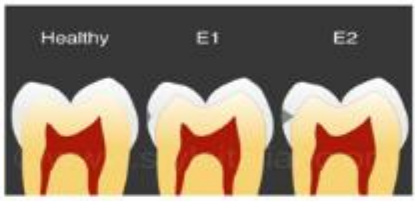

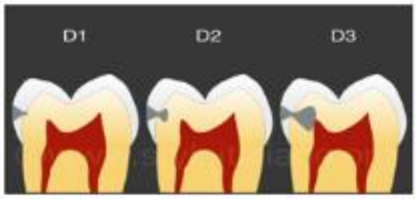

Recommendations for anterior adult teeth:

- For small carious lesions not yet spreading into dentin, we typically recommend PVP-I to avoid staining in the esthetic region of the mouth.
- Good OH and adequate use of fluoride is recommended to prevent progression & necrosis. Consider prescription toothpaste.
- Curodont is an option; evidence is emerging.
- Caries that is spreading into dentin can be treated with traditional restorations vs. PVP-I vs. SDF, depending on patient/guardian preference. Typically SDF on anterior permanent teeth is reserved for very high risk patients at risk for losing their dentition at an early age.
- Necrotic permanent anterior teeth require root canal treatment or extraction.

Discussing Treatment Options

All treatment options should be given to parents.

All treatment options include:

- No treatment
- Fluoride Varnish
- Povidone Iodine
- Curodont
- Silver Diamine Fluoride
- Protective Restoration
- SMART Filling

- Traditional Filling – materials include G.I vs. Resin
- Crown – SSC vs. Zirconia
- Extraction
- Nitrous Oxide (to use with decisions above)
- Sedation (to use with decisions above)

Some treatment options do not fit in with our philosophy. (e.g. sedating a 2 year old for dental treatment/ using zirconia crowns – regardless – all options are explained & rationale for recommendations.)

Dictation of Treatment Options

Template in blue to include on RECARE notes:

Discussed risks of no treatment including pain, abscess, damage to permanent dentition. Discussed risk of progression of caries when using medical management.

Active Surveillance

In cases when parents elect to monitor primary necrotic teeth because they are asymptomatic, we Code these in Open Dental. (This is useful for research purposes & communication).

The Quick Button ("**A**") is used. This stands for Active Surveillance.

The following auto-note should be used. "**Active Surveillance**": "Discussed presence of necrotic primary tooth and that nerve of tooth is likely dead. Explained that this can be a source of infection that may or may not remain localized and could potentially cause damage to the permanent successor. Provided option of EXT vs Active Surveillance. POC elected for Active Surveillance at this time due to the absence of nocturnal/spontaneous pain or difficulty eating/drinking/sleeping. Reviewed that tooth may become symptomatic in the future, and if so, encouraged follow-up with dental team. POC expressed understanding of RBA."

Follow-up Care

Typical follow-up is 6 months.

Tighter RECARE is warranted in higher-risk children/ when disease is rapidly-progressing.

1-3 month follow-up may be recommended to monitor progression clinically/ radiographically.

Consider for ICDAS 2+ (Anterior Permanent) and (Posterior Primary and Permanent).
